# Supplementary material for: Visualizing moiré ferroelectricity via plasmons and nano-photocurrent in graphene/twisted-WSe2 structures
Source: Nat Commun. 2023 Oct 4;14:6200. doi: 10.1038/s41467-023-41773-x (PMC10550968; doi:10.1038/s41467-023-41773-x)
Supplement: Supplementary file 1 — Supplementary Information [file 41467_2023_41773_MOESM1_ESM.pdf]

Supplementary information for "Visualizing moiré ferroelectricity via plasmons and nano-photocurrent in graphene/twisted-WSe_2 structures"

Shuai Zhang[1]†*, Yang Liu[2]†, Zhiyuan Sun[3,8], Xinzhong Chen[1,4], Baichang Li[2], S. L. Moore[1], Song Liu[2], Zhiying Wang[2], S. E. Rossi[1], Ran Jing[1], Jordan Fonseca[5], Birui Yang[1], Yinming Shao[1], Chun-Ying Huang[6], Taketo Handa[6], Lin Xiong[1], Matthew Fu[1], Tsai-Chun Pan[1], Dorri Halbertal[1], Xinyi Xu[2], Wenjun Zheng[4], P.J. Schuck[2], A.N. Pasupathy[1], C.R. Dean[1], Xiaoyang Zhu[6], David H. Cobden[5], Xiaodong Xu[5], Mengkun Liu[4], M.M. Fogler[7], James C. Hone[2], D.N. Basov[1]*

[1]Department of Physics, Columbia University, New York, NY, 10027, USA

[2]Department of Mechanical Engineering, Columbia University, New York, NY, 10027, USA

[3]Department of Physics, Harvard University, Cambridge, MA, 02138 USA.

[4]Department of Physics and Astronomy, Stony Brook University, Stony Brook, NY, 11794 USA.

[5]Department of Physics, University of Washington, Seattle, WA, 98195, USA

[6]Department of Chemistry, Columbia University, New York, NY, 10027, USA

[7]Department of Physics, University of California, San Diego, La Jolla, CA, 92093 USA

[8]Current address: State Key Laboratory of Low-Dimensional Quantum Physics and Department of Physics, Tsinghua University, Beijing 100084, P.R. China

†These authors contributed equally: Shuai Zhang, Yang Liu

* Corresponding email: sz2822@columbia.edu; db3056@columbia.edu

This file includes:

Supplementary Notes

Supplementary note 1: Two approaches to quantifying the carrier density in graphene with s-SNOM

Supplementary note 2: Device characterization

Supplementary note 3: Carrier density dependence of the scattering signal

Supplementary note 4: Plasmon excitation in R-stacking WSe_2 bilayers.

Supplementary note 5: Theory of ferroelectric doping

Supplementary note 6: Electrical field from the ferroelectric domains

Supplementary note 7: Regime with plasmon propagation

Supplementary note 8: Photon energy dependence of the ferroelectric modulated plasmonic response

Supplementary note 9: Interpretation of the sinusoidal shape of the near-field profile across domains

Supplementary note 10: Quality of the layer interfaces and their effects on ferroelectricity-induced doping

Supplementary note 11: Electron cooling length in photocurrent measurement

Supplementary note 12: Near-field photocurrent simulations

Supplementary Figures.

Supplementary References

Supplementary note1: Two approaches to quantifying the carrier density in graphene with s-SNOM

There are two approaches to quantifying the carrier density in graphene using s-SNOM data. One is to extract the carrier density from the period of the propagating plasmon polariton[1,2], and another is based on the near-filed amplitude or phase evolution[3]. Now we discuss these two approaches. We note that the second approach is more suitable for samples with inhomogeneous doping, such as the samples with moiré patterns in this work.

(1) Approach 1: to extract the carrier density of graphene from the plasmon polariton period

In two-dimensional electron systems, the plasmon can be excited by a photon with proper momentum. With s-SNOM, whose tip can impart momentum, the plasmon excitations can be measured. Once the tip momentum is in resonance with the plasmon polariton momentum, the propagating plasmon polariton can be formed, manifesting in fringes in the real space s-SNOM image. The plasmon polariton obeys the scaling law $\omega_p \propto q^{1/2}n^{1/4}$, where ω_p is the plasmon energy, q is the momentum, and n is the carrier density[4]. The momentum q can be read from the plasmon propagating period λ ($q = \frac{2\pi}{\lambda}$). Therefore, for a given photon energy, the carrier density information can be extracted from the plasmon polariton period.

We note that for a device with multilayer materials, the scaling law would be modified by the device structure and the dielectric functions of these materials. However, the resultant scaling law can be readily obtained by solving the Fresnel reflection of p-polarized light (r_p calculations).

It is also noteworthy that approach 1 is only suitable for the cases with homogenous carrier doping. For a device with carrier density inhomogeneity, the boundaries of the electron/hole puddles can launch and reflect the plasmon polariton, thereby forming complex plasmon propagating patterns. The extracted plasmon period and the corresponding carrier density would be averaged ones.

(2) Approach 2: to extract the doping from near-field scattering amplitude or phase contrast

As discussed above, for a sample with moiré patterns, we cannot use the propagating plasmon polariton to extract the carrier density in each domain. Now we should focus on the near-field scattering amplitude or phase. In this work, the near-field amplitude was measured. For graphene, the scattering amplitude is a function of carrier density. Therefore, we can first obtain the relationship between the near-field amplitude and the carrier density, and then use this established relationship to get the carrier density information.

In this work, we are interested in the doping difference between the graphene above AB and the graphene BA ferroelectric domains. Thus, by comparing the near-field amplitude of graphene above these two domains, an accurate carrier density can be extracted. To obtain the carrier density, we need to know the charge density point of each domain and the geometric capacitance of the device.

1. To get the charge density point (CNP):

We recorded the near-field amplitude, s_4, on each type of domain when the backgate voltage, V_g, was swept. Namely, we obtained s_4 versus V_g on two domains. The CNPs of the two types of domains correspond to the peak positions of s_4, in the s_4 versus V_g curves. We denote the CNPs of the AB and BA domains as V_{g-AB}, and V_{g-BA}, respectively.

2. To calculate the carrier density of each domain:

a) The carrier density of the AB domain at backgate voltage V_g is $n_{AB} = \frac{C}{e}\left(V_g - V_{g-AB}\right)$, where C is the geometric capacitance.;

b) The carrier density of the BA domain at backgate voltage V_g is $n_{BA} = \frac{C}{e}\left(V_g - V_{g-BA}\right)$;

c) The carrier density difference between the AB and BA domains is $\Delta n = \frac{C}{e}(V_{g-AB} - V_{g-AB})$. This carrier density difference originates from ferroelectricity.

We emphasize that we should use the doping regimes without propagating/reflected plasmon polariton to analyze the carrier density. In the plasmon resonance regime, the measured near-field signal would be contributed to by the propagating plasmon polariton launched from nearby domains. In this case, the near-field amplitude is not exclusively from the local domain, and a carrier density error might arise.

Supplementary note 2: Device characterization

Device A

The structure of Device A is shown in Supplementary Figure 1. The contact configuration used for the photocurrent is illustrated. Contact 1 is grounded, and contact 2 is connected to a preamplifier for photocurrent measurements. Before the near-field experiments, the sample is characterized by piezoresponse force microscopy (PFM).

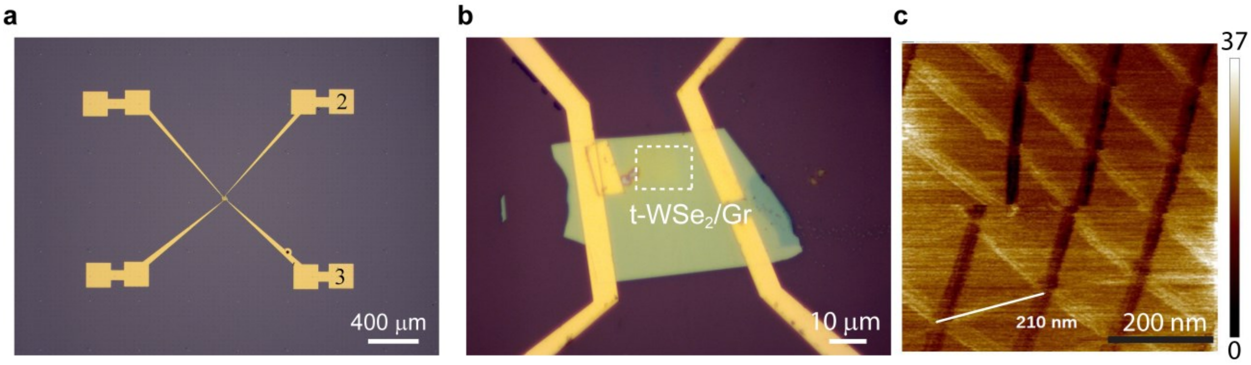

Supplementary Figure 1| Device A a,b, Optical microscope image of device-A. The gold contacts are numbered. Contact 1 is grounded. Contact 2 is connected to a preamplifier for photocurrent measurements. The position of t-WSe_2/graphene is marked by the dash lines. **c**, The

phase image of piezoresponse force microscopy (PFM) on the R-stacking WSe_2/graphene device. The triangular domains are observed.

Device B

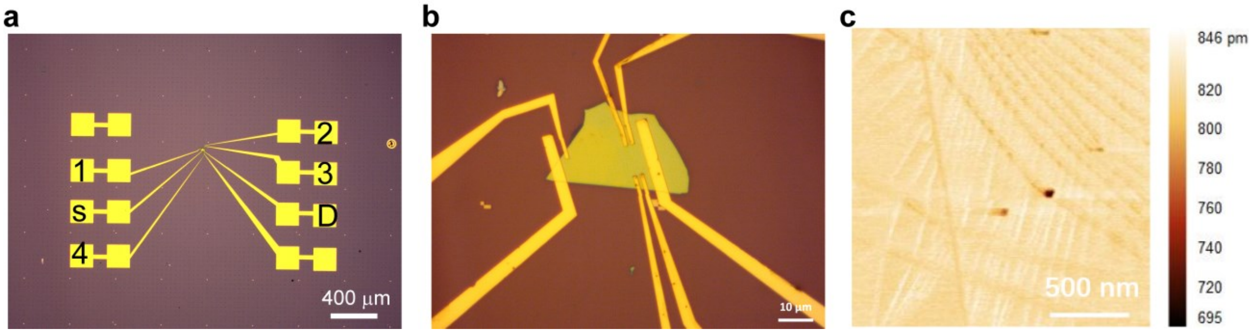

Supplementary Figure 2| Device B a,b, Optical microscope image of Device-B. The gold contacts are numbered. Contact "S" is grounded. Contact "D" is connected to a preamplifier for photocurrent measurements. **c**, The amplitude image of piezoresponse force microscopy (PFM) on the t-WSe_2/graphene device. The domain boundaries are observed.

Supplementary note 3: Carrier density dependence of the scattering signal

3.1 Extended data on the near-field signal as a function of back-gate voltage

Here, we show the near-field scattering amplitude images acquired at various back gate voltages.

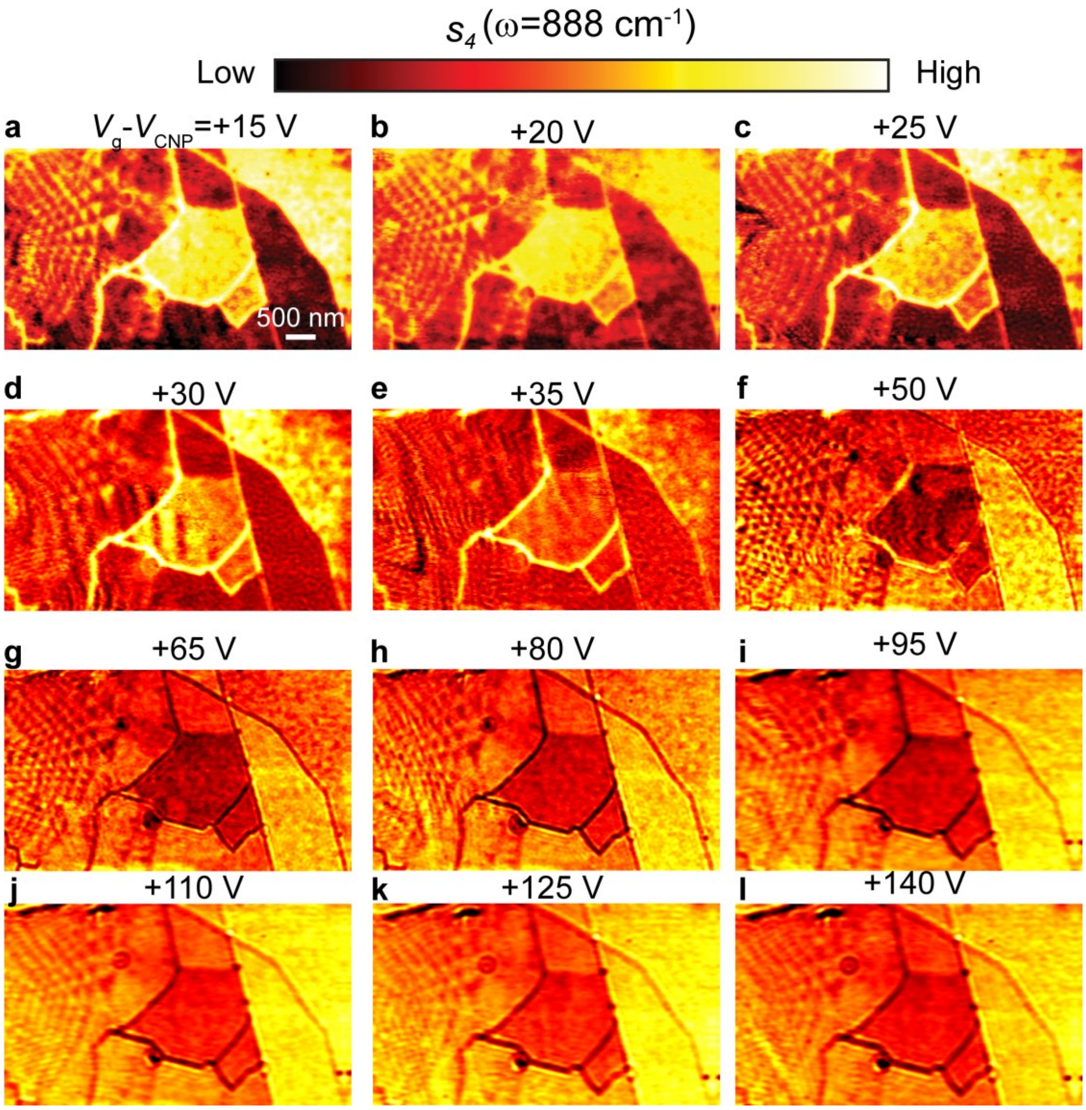

Supplementary Figure 3| Near-field scattering amplitude images acquired at various back gate voltages, $V_g - V_{CNP}$. All the data were acquired on Device A.

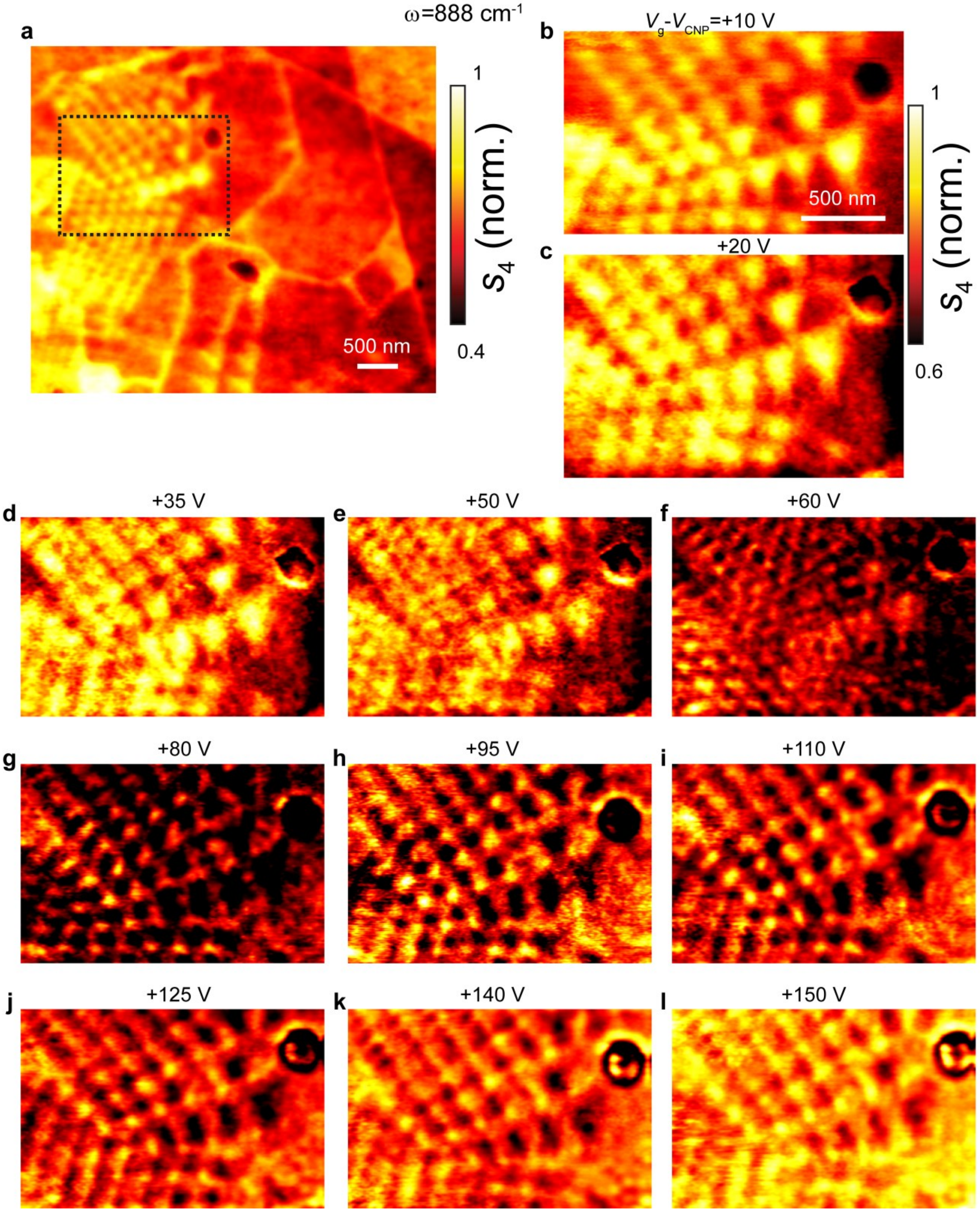

Supplementary Figure 4| High-resolution near-field scattering amplitude images acquired at various back gate voltages for Device A. To make the contrast between domains clear, the amplitude in each image is normalized to the maximum scattering in the view.

3.2 Analytical result of the near-field signal

Before using the light-rod model to numerically calculate the near-field signal, we first use the simplified model to provide an intuitive analytical result on the near-field signal evolution.

The tip-sample coupling G is determined by the tip momentum weighting function $w(k)$ and momentum-dependent Fresnel reflection coefficient r_p. That is,

$$G \approx \int w(k) r_p(k,\omega) dk \tag{1}$$

The sample has three layers: the WSe_2 bilayer on the top, graphene, and dielectric (h-BN, SO_2 on Si) on the bottom. To simplify the calculation of r_p, we assume that the bottom dielectric is thick h-BN and only consider the energy range without phonon resonance from h-BN. Then, the reflection structure is vacuum (labeled by 0)/WSe_2 of thickness t (labelded by 1)/graphene on thick h-BN (labeled by 2). The vacuum, WSe_2, and h-BN permittivities are ε_0, ε_1 and ε_2, respectively. For WSe_2 and h-BN, the in-plane and the out-of-plane permittivity are not the same, so ε_1, ε_2 are effective permittivity, are denoted by $\varepsilon_j = \sqrt{\varepsilon_{jt}\varepsilon_{jz}}$, where j=1 or 2, ε_{jt}, ε_{jz} are in-plane and out-of-plane permittivity, respectively. The effective confinement factor is $\eta = \sqrt{\frac{\varepsilon_{jt}}{\varepsilon_{jz}}}$.

The momentum-dependent Fresnel reflection is,

$$r_p(q,\omega) = \frac{r_{01} + r_{12} e^{2ik_{z1}t}}{1 + r_{01} r_{12} e^{2ik_{z1}t}} \tag{2}$$

with

$$r_{01} = \frac{\varepsilon_1 k_{z0} - \varepsilon_0 k_{z1}}{\varepsilon_1 k_{z0} + \varepsilon_0 k_{z1}} \tag{3}$$

$$r_{12} = \frac{\frac{\varepsilon_2^t}{k_{z2}} - \frac{\varepsilon_1^t}{k_{z1}} + \frac{\sigma_S}{\omega}}{\frac{\varepsilon_2^t}{k_{z2}} + \frac{\varepsilon_1^t}{k_{z1}} + \frac{\sigma_S}{\omega}} \tag{4}$$

Here $k_{zj} = \sqrt{\varepsilon_j \left(\frac{\omega}{c}\right)^2 - q^2}$, j=0, 1, 2, which correspond to vacuum, WSe_2, and h-BN.

In the quasi-electrostatic limit ($k \gg \frac{c}{\omega}$), for all j = 0, 1, 2, we can make the approximation, $k_{zj} \approx iq$.

$$r_{01} \approx \frac{\varepsilon_1 - \varepsilon_0}{\varepsilon_1 + \varepsilon_0} \tag{5}$$

$$r_{12} \approx \frac{\varepsilon_2-\varepsilon_1+\frac{\sigma_S}{\omega}(iq)}{\varepsilon_2+\varepsilon_1+\frac{\sigma_S}{\omega}(iq)} = \frac{\frac{\varepsilon_2-\varepsilon_1}{\varepsilon_2+\varepsilon_1}-\frac{\sigma_S q}{i(\varepsilon_2+\varepsilon_1)\omega}}{1-\frac{\sigma_S q}{i(\varepsilon_2+\varepsilon_1)\omega}} \tag{6}$$

$$r_p(q,\omega) = \frac{\frac{\varepsilon_1-\varepsilon_0}{\varepsilon_1+\varepsilon_0}+\frac{\frac{\varepsilon_2-\varepsilon_1}{\varepsilon_2+\varepsilon_1}-\frac{\sigma_S q}{i(\varepsilon_2+\varepsilon_1)\omega}}{1-\frac{\sigma_S q}{i(\varepsilon_2+\varepsilon_1)\omega}}e^{-2\eta qt}}{1+\frac{\varepsilon_1-\varepsilon_0}{\varepsilon_1+\varepsilon_0}\frac{\frac{\varepsilon_2-\varepsilon_1}{\varepsilon_2+\varepsilon_1}-\frac{\sigma_S q}{i(\varepsilon_2+\varepsilon_1)\omega}}{1-\frac{\sigma_S q}{i(\varepsilon_2+\varepsilon_1)\omega}}e^{-2\eta qt}} \tag{7}$$

1) At the low doping level and the given excitation energy, the plasmon resonance momentum is much larger than the tip momentum, $\frac{\sigma_S q}{i(\varepsilon_2+\varepsilon_1)\omega} \ll 1$.

$$\begin{aligned} r_p(q,\omega) &\approx \frac{\frac{\varepsilon_1-\varepsilon_0}{\varepsilon_1+\varepsilon_0}+\left(\frac{\varepsilon_2-\varepsilon_1}{\varepsilon_2+\varepsilon_1}-\frac{\sigma_s q}{i(\varepsilon_2+\varepsilon_1)\omega}\right)e^{-2\eta qt}}{1+\frac{\varepsilon_1-\varepsilon_0}{\varepsilon_1+\varepsilon_0}\left(\frac{\varepsilon_2-\varepsilon_1}{\varepsilon_2+\varepsilon_1}-\frac{\sigma_s q}{i(\varepsilon_2+\varepsilon_1)\omega}\right)e^{-2\eta qt}} \\ &\approx \frac{\frac{\varepsilon_1-\varepsilon_0}{\varepsilon_1+\varepsilon_0}+\left(\frac{\varepsilon_2-\varepsilon_1}{\varepsilon_2+\varepsilon_1}\right)e^{-2\eta qt}}{1+\frac{\varepsilon_1-\varepsilon_0}{\varepsilon_1+\varepsilon_0}\left(\frac{\varepsilon_2-\varepsilon_1}{\varepsilon_2+\varepsilon_1}\right)e^{-2\eta qt}} + \frac{\frac{1}{(\varepsilon_2+\varepsilon_1)}e^{-2\eta qt}}{1+\frac{\varepsilon_1-\varepsilon_0}{\varepsilon_1+\varepsilon_0}\left(\frac{\varepsilon_2-\varepsilon_1}{\varepsilon_2+\varepsilon_1}\right)e^{-2\eta qt}}\chi_g \end{aligned} \tag{8}$$

The real permittivity decreases as the carrier density increases, as shown in Supplementary Figure 5. It should be noted that the peak in the real permittivity at $2\omega = \mu$ is smeared out by the damping[5-8]. At low temperatures, this peak could appear. So $Re(r_p(q,\omega))$ decreases when the Fermi energy is tuned away from the charge neutrality point, thus resulting in the decrease in the near-field scattering amplitude.

2) However, when the carrier density is high enough, the plasmon momentum will shift towards the tip momentum, eventually overlapping. Then the near-field amplitude will increase, as shown in Supplementary Figure 6.

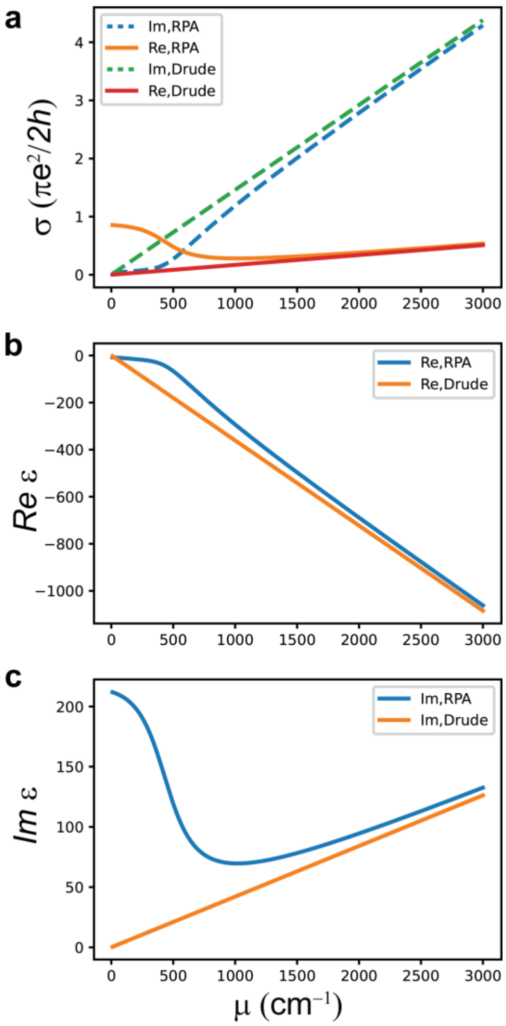

Supplementary Figure 5| The optical conductivity and permittivity of graphene with a photon energy of 860 cm^{-1}. Top: Conductivity of graphene with a photon energy of 860 cm^{-1} as a function of Fermi energy. The Drude response only considers the intraband transition, whereas the random phase approximation (RPA) includes both the intraband and interband transitions. Middle: The real permittivity of graphene as a function of Fermi energy. Bottom: The imaginary permittivity of graphene as a function of Fermi energy. The relaxation rate used here is 100 cm^{-1}.

3.3 Numerical simulations of the near-field signal

The near-field signals are simulated using the light rod model[9]. With doping, the near-field amplitude near $\omega = 860\ cm^{-1}$ first decreases and then increases, which is consistent with the experimental results. The Fresnel reflection coefficient is calculated using the transfer matrix method. We find that the plasmonic resonance, which corresponds to a peak of $Im(r_p)$, gradually shifts toward lower momentum with increasing doping. At a specific doping, the plasmon momentum overlaps the momentum imparted by the tip.

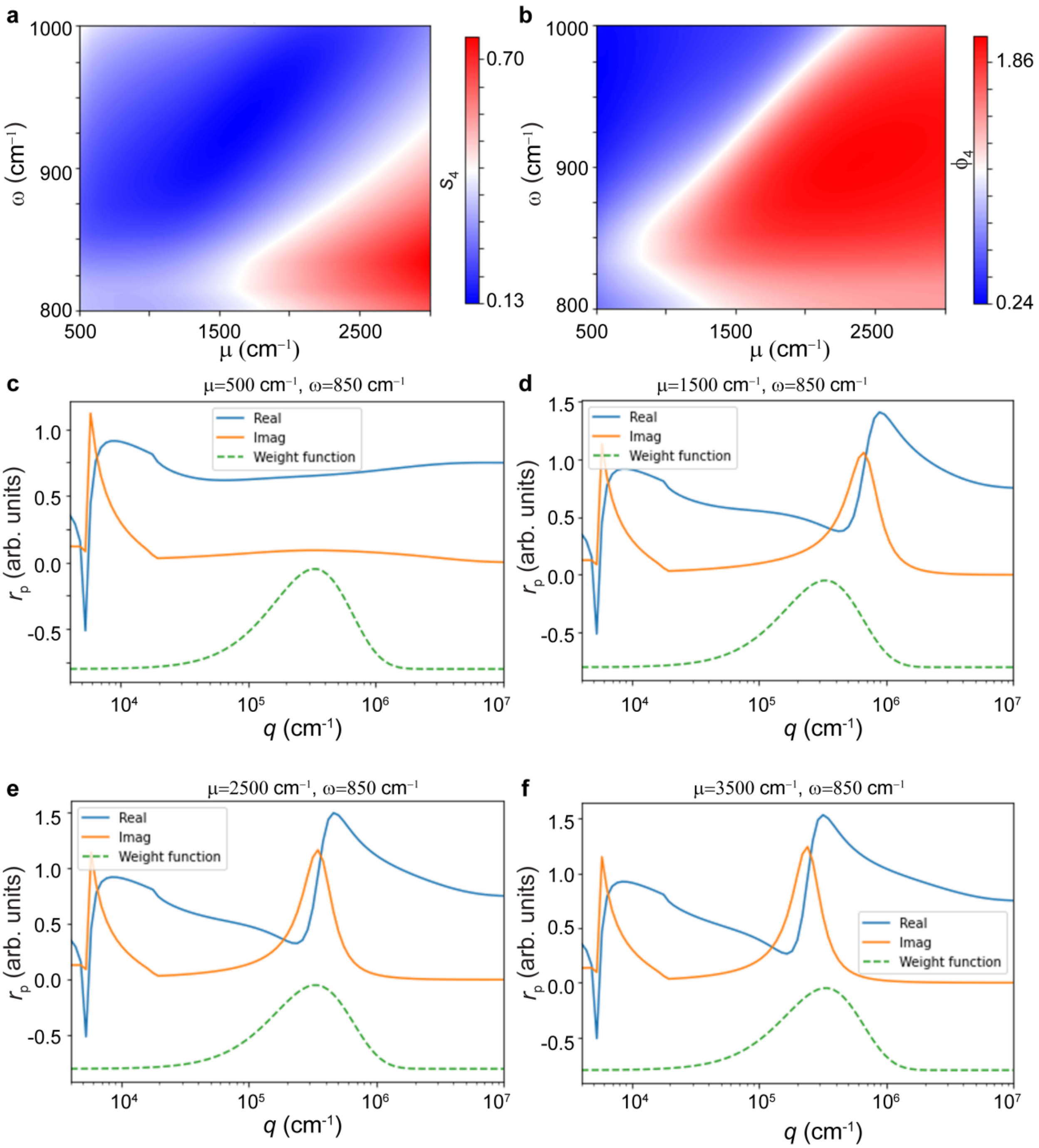

Supplementary Figure 6| Numerically calculated near-field scattering signal. a,b, Near-field scattering amplitude (**a**) and phase (**b**) as a function of photon energy and chemical potential. The device geometry is the same as that of Device A, and the light rod model is used. **c-f** Fresnel reflection coefficient at a series of chemical potentials. The photon energy $\omega = 850\ cm^{-1}$. The blue dashed lines denote the near-field coupling weight function.

Supplementary note 4: Plasmon excitation in R-stacking WSe_2 bilayers.

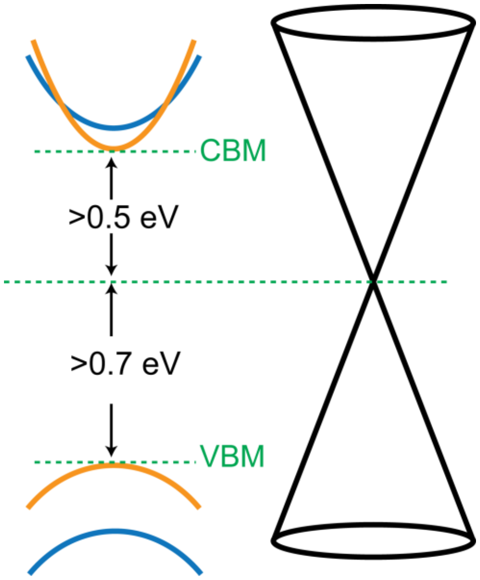

Supplementary Figure 7| Band alignment in the WSe_2-graphene heterostructure. In our experiment, the maximum Fermi energy of the graphene is less than 0.5 eV. Therefore, the back gate doped carrier will enter in the graphene and cannot be transferred into the WSe_2.

The WSe_2 is not doped by the back gate in our experimental back gate voltage range. This conclusion is supported by the band alignment between the WSe_2 and graphene, as shown in Supplementary Fig. 7. The energy difference between the graphene Dirac point and the WSe_2 band edges is larger than 0.5 eV[10]. In our experiments, the maximum graphene Fermi energy is 0.36 eV. Therefore, the graphene Fermi energy is in the bandgap of WSe_2. The carriers from the back gate can only enter into graphene. Therefore, the observed near-field signal is dominated by the plasmonic excitation of graphene; the plasmonic excitation of WSe_2, if it is unintentionally doped, is negligible. This conclusion is confirmed by both simulations and control experiments discussed below.

To compare the plasmonic excitations of graphene and WSe_2, we first performed calculations of their plasmonic dispersions. To this end, we assume that both graphene and each layer of WSe_2 are doped with the same carrier density, which is $5 \times 10^{12}\ cm^{-2}$. The parameters used to calculate the optical conductivity are listed here: effective mass of WSe_2 $m_e = 0.5m_0$, damping rate of WSe_2 carrier $\gamma = 200\ cm^{-1}$, and damping rate of graphene carrier $\gamma = 20\ cm^{-1}$. In the simulations, all the substrate layers are included, which are 58 nm h-BN/285 nm SiO_2/Si. The calculated plasmonic dispersions are shown in Supplementary Fig. 8. From the dispersions, we can find that the plasmonic excitation of the WSe_2 bilayer exists below 500 cm^{-1}, which is far away from the photon energy used in our experiments (860~920 cm^{-1}). In contrast, strong graphene plasmon modes appear in the energy regimes of our experiments. The extra models around 800 cm^{-1} and 1500 cm^{-1} are from the phonon polaritons of thin h-BN substrate flakes[11].

The different plasmonic behaviors of WSe_2 and graphene originate from their distinct energy dispersions. For WSe_2, the energy dispersion is parabolic, whereas the graphene shows linear energy dispersion. As a result, they exhibit different Drude weight formulas and plasmon dispersion. For WSe_2, the Drude weight is $D = \frac{\pi e^2 n}{m}$, whereas graphene's Drude weight is $D = \frac{e^2 v_F \sqrt{\pi n}}{\hbar}$, where n is carrier density, m is effective mass of carrier, $\hbar$ is reduced Planck constant, and v_F is Fermi velocity[12].

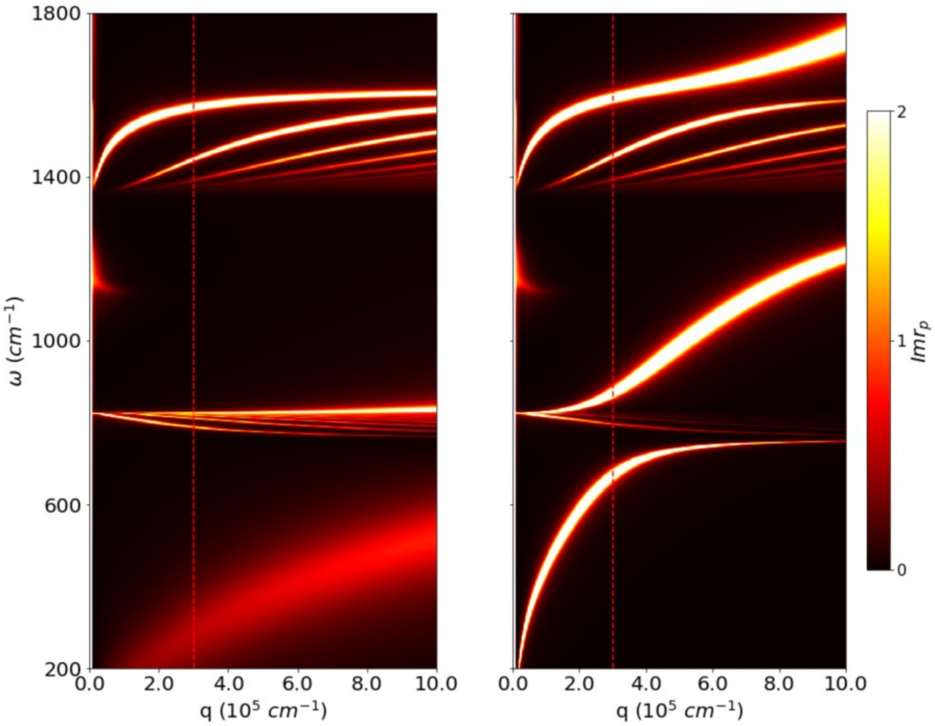

Supplementary Figure 8| Calculated dispersion of the plasmon polariton. The dispersions are visualized using a false-color map of the imaginary part of the reflection coefficient of p-polarized light ($Im(r_p)$). The red dashed line indicates the momentum at which the tip can strongly couple with the polaritons. Left panel: The dispersion of doped double-layer WSe_2. Right panel: The dispersion of doped double-layer WSe_2/graphene. These two stacks are put on 58 nm h-BN/280 nm SiO_2. Graphene and each WSe_2 layer are doped with a carrier density of 5×10^{12} cm^{-2}.

To further verify that the observed moiré contrast originates from the ferroelectric modulated plasmon response in graphene, we performed control experiments on a sample without graphene. The sample structure is the same as that in Devices A and B, except for excluding a graphene layer. The sample structure is shown in Supplementary Figure 9a. The back gate electrode is a graphite layer, and the gating dielectric is a h-BN flake with a thickness of 40 nm. No noticeable near-field amplitude evolution was observed in WSe_2 with carrier density doped up to 1.3×10^{13} cm^{-2} (Supplementary Fig. 9 d,e).

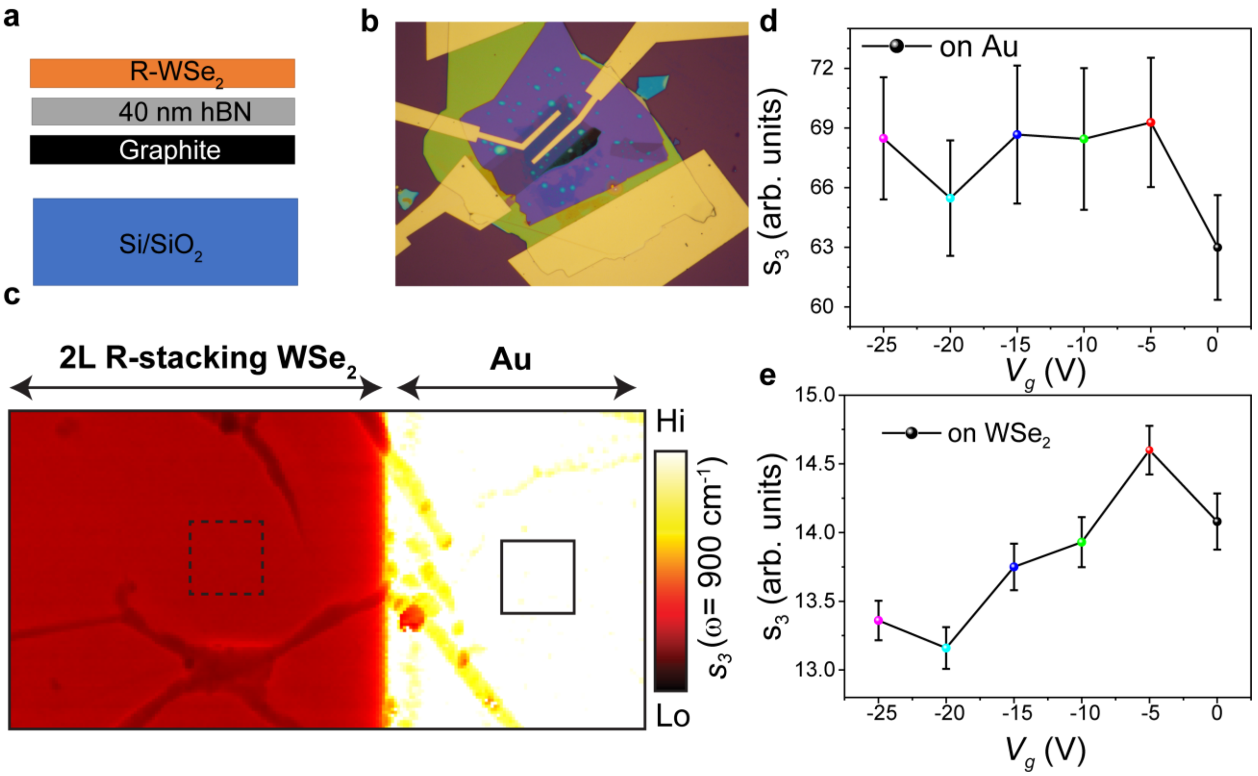

Supplementary Figure 9| Plasmon response of R-stacking WSe_2 bilayer. a, Schematic of the device structure. **b**, Optical micrograph of the device. **c**, Near-field amplitude images. The left side is WSe_2, and the right side is a gold pad used to renormalize the near-field signal. The image was acquired using a CO_2 laser with photon energy ω=900 cm^{-1}. **d,e,** Near-field amplitude signal at various back gate voltages for gold and WSe_2. To acquire the data, we first obtained a series of near-field scattering amplitude images, like **c**, at various back gate voltages. Then, each data point in **d** and **e** is obtained by averaging the signals in the areas delineated by squares in **c**. No noticeable plasmonic response is observed. The root-mean-square-deviation is calculated by $\sigma = \sqrt{\frac{1}{N}\sum_{n=1}^{N}(s_{3n} - \bar{s_3})^2}$, where N, s_{3n}, and $\bar{s_3}$ are number of pixels, near-field amplitude of each pixel, and the mean value of near-field amplitude.

Supplementary Note 5: Theory of ferroelectric doping

5.1 General Remarks

The electrical potential immediately above a 2D plane made of 'ferroelectric' moiré superlattices can be well approximated by

$$\phi_f(\boldsymbol{r}) = \phi_0 \begin{cases} 1 & \boldsymbol{r} \text{ in AB domain} \\ -1 & \boldsymbol{r} \text{ in BA domain} \end{cases} \tag{9}$$

due to lattice relaxation, the domain walls are much thinner than the domain period. Away from the 2D plane, this potential decays with a decay length on the order of the moiré period. The periodic ferroelectric potential causes doping of graphene placed parallel to the plane of moiré superlattices. The resulting local Fermi energy $\mu(\boldsymbol{r})$ of graphene is determined by

$$\mu + \phi_f + \phi[\rho(\mu)] = V_g \tag{10}$$

where $\phi(\boldsymbol{r}) = \int d\boldsymbol{r}' \frac{1}{\epsilon}\left(\frac{1}{|\boldsymbol{r}-\boldsymbol{r}'|} - \frac{1}{\sqrt{4d^2+|\boldsymbol{r}-\boldsymbol{r}'|^2}}\right)\rho(\boldsymbol{r}')$ is the screening electrical potential due to the doped charge on graphene, $\rho = \frac{1}{\pi}\frac{\mu^2}{v_F^2\hbar^2}\mathrm{Sign}[\mu]$ is the local charge density, V_g is the gate voltage, and d is the distance of graphene to the gate.

In the simple case of a stripe moiré lattice, the potential has the analytical form:

$$\phi_f(x,z) = \phi_0 \frac{2}{\pi} \mathrm{ArcTan}\left(\frac{\sin\frac{2\pi}{L}x}{\sinh\frac{2\pi}{L}h}\right) \tag{11}$$

where L is the strip period, and h is the distance away from the 2D plane. If the screener is a perfect metal, the screening charge is

$$\rho(x) = \phi_0 \frac{2}{\pi L} \frac{\sin\left(\frac{2\pi}{L}x\right)\cosh\left(\frac{2\pi}{L}h\right)}{\sinh^2\left(\frac{2\pi}{L}x\right)+\sin^2\left(\frac{2\pi}{L}h\right)} \tag{12}$$

5.2 Estimations by single Fourier component

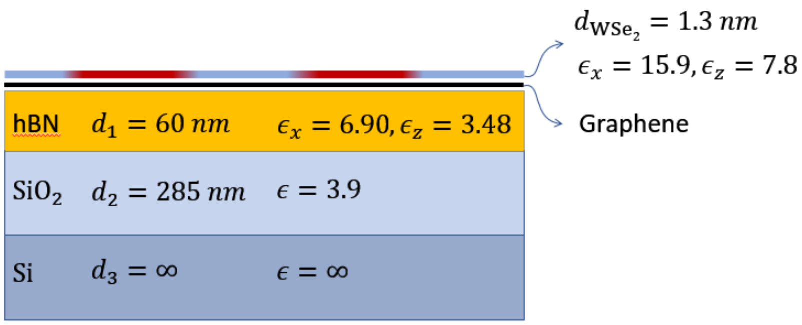

Supplementary Figure 10| Schematics of the device.

For the device in Supplementary Fig. 10, where the moiré ferroelectric is on top, the resulting screening charge in graphene may be represented as a doping chemical potential satisfying:

$$\frac{1}{\epsilon_{\text{eff}}}\frac{\mu^2}{\varepsilon_L} + \mu = f\phi_f, \tag{13}$$

$$\mu = \frac{\epsilon_{\text{eff}}\varepsilon_L}{2}\left(-1 + \sqrt{1 + \frac{4f\phi_f}{\epsilon_{\text{eff}}\varepsilon_L}}\right) \to \begin{cases} f\phi_f & \text{if } \phi_f \ll \frac{\epsilon_{\text{eff}}\varepsilon_L}{4f} \\ \sqrt{\epsilon_{\text{eff}} f \varepsilon_L \phi_f} & \text{if } \phi_f \gg \frac{\epsilon_{\text{eff}}\varepsilon_L}{4f} \end{cases} \tag{14}$$

where $\epsilon_{\text{eff}} = \frac{1-R_{\text{top}}R_{\text{bottom}}}{(1+R_{\text{top}})(1+R_{\text{bottom}})}$ is the effective 2D dielectric environment for graphene at wave vector q and $f = 2\frac{1+R_{\text{bottom}}}{2+R_{\text{WSe2}}+R_{\text{bottom}}}$ is a screening factor for the ferroelectric potential beneath WSe_2 (Ref. [13]). The R_{top} (R_{bottom}) is the reflection coefficient for the electrostatic potential at the top (bottom) side of the 2D graphene-ferroelectric system. Without any screening layers above WSe_2, given a moiré period of 340 nm, one has $\varepsilon_L = 2.8$ meV, $\epsilon_{\text{eff}} = 3.1$ and $f = 0.51$. Therefore, $\phi_f \approx 56$ meV $\gg \frac{\epsilon_{\text{eff}}\varepsilon_L}{4f}$ and the doping level is about $\sqrt{\epsilon_{\text{eff}} f \varepsilon_L \phi_f} \approx 16$ meV. (Adding a 20 nm water layer would boost the screening to $\epsilon_{\text{eff}} = 16$ and $f = 1.7$, and the doping level to $\sqrt{\epsilon_{\text{eff}} f \varepsilon_L \phi_f} \approx 65$ meV.)

From the experiment, we see that the voltage needed to cancel the doping in a domain is about $V = 6\,V$. Given the capacitance $\frac{1}{C_g} = 4\pi e\left(\frac{d_1}{\epsilon_z} + \frac{d_2}{\epsilon}\right)$ of the device, this voltage corresponds to a doping density of $n = 3.7 \times 10^{11}$ cm^{-2}, considering the thickness $d_1 = 60$ nm and out-of-plane dielectric $\epsilon_z = 3.48$ for h-BN and the thickness $d_2 = 285$ nm and dielectric $\epsilon = 3.9$ for SiO_2. This density corresponds to a doping level of 71 meV. To explain such a large doping level, one needs to assume that the ferroelectric potential is $\phi_f \sim 1.1$ eV.

5.3 Effect of the water layer on ferroelectricity-induced doping

In addition, we also performed theoretical calculations to show the effects of potential contaminations on ferroelectricity-induced doping. We took water as an example since water is a prototype polar molecule.

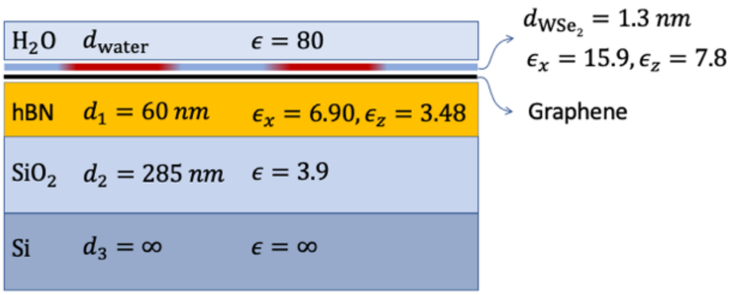

Supplementary Figure 11| Schematics of the devices with water.

In this section, we show the numerically exact doping levels computed from the linear screening model. This approach is justified because $\phi_f \gg \frac{\epsilon_{\mathrm{eff}}\varepsilon_L}{4f}$ such that the "quantum capacitance" of graphene can be neglected.

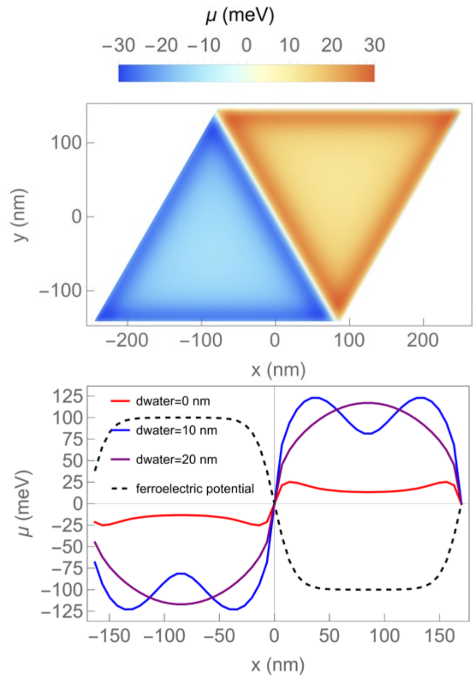

Supplementary Figure 12| The effect of water on ferroelectric-induced doping. Top: The doping profile of graphene in the device in Supplementary Figure 11. The bare ferroelectric potential is $\phi_f = 56$ meV, the moiré period is $L = 340$ nm, and the thickness of the water layer is 0. Bottom: the horizontal line-cut of the top figure at $y = 0$, with three different thicknesses of the water layer. Note that the experimentally obtained value of the doping is 71 meV.

5.4 Numerical Results for the nonlinear screening problem

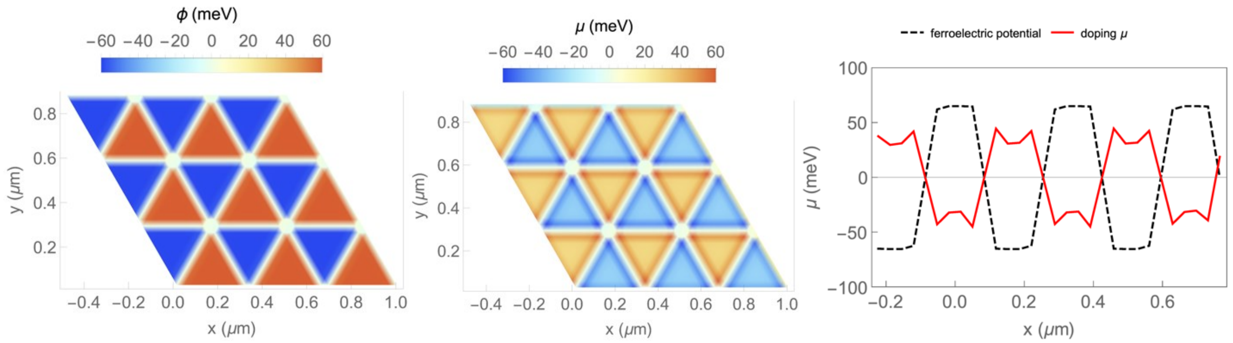

Supplementary Figure 13| Numerical results with a grid of 30 × 30. Left: The ferroelectric potential (about 66 meV) of twisted WSe_2. The moiré period (length of a lattice vector) is 340 nm. The distance between the graphene and the gate is 340 nm. The dielectric of the environment is $\epsilon = 5$. Middle: The Fermi energy of graphene on top of WSe_2. Right: Line-cut of the middle panel at $y = 0.43\ \mu m$.

5.5 Charge transfer due to in-gap states of WSe_2

We assume there are some immobile electronic states, with a density of states D, at energies inside the gap of WSe_2 such that there is charge transfer between WSe_2 and graphene. Experimentally, bare WSe_2 is slightly n-doped. Therefore, there is probably electron transfer from WSe_2 to graphene. These in-gap states have wavefunctions evenly distributed among the two WSe_2 layers, and therefore they are unaffected by the ferroelectric potential. Since electrons in graphene experience the ferroelectric potential, the charge transfer between graphene and WSe_2 will be inhomogeneous, alternating across AB and BA domains. The resulting local doping level μ in graphene is:

$$\mu - \mu_0 + \frac{\delta n(\mu)}{C} + \frac{\delta n(\mu)}{D} = \phi_f\,, \quad \frac{n_0(\mu_0) - n_t}{C_g} = V_g, \tag{15}$$

where ϕ_f is the ferroelectric potential, μ_0 is the chemical potential assuming no ferroelectric potential, $\delta n = \frac{1}{\pi\hbar^2 v_F^2}(\mu^2 - \mu_0^2)$ is the density of transferred charge due to the ferroelectric potential, $C = \frac{1}{4\pi e^2 d}$ and d are the geometric capacitance, and the distance between graphene and the central plane of WSe_2, respectively, n_t is the charge density in graphene at zero gate voltage, and $1/C_g = \frac{e}{4\pi}\left(\frac{d_1}{\epsilon_z} + \frac{d_2}{\epsilon}\right)$ is the inverse of the capacitance between graphene and the back gate. The charge transfer compensates for the ferroelectric potential in two ways: generating the

electrostatic potential $\frac{\delta n(\mu)}{C}$ and the chemical potential difference $\mu - \mu_0 + \frac{\delta n(\mu)}{D}$. The electrostatic potential $\frac{\delta n(\mu)}{C}$ is generated by the local dipole density between the graphene layer and the WSe_2 layer and is much weaker than that generated by a charge modulation $\delta n(\mu)$ only on graphene. Therefore, the same ferroelectric potential leads to a large doping level on graphene.

The solution is

$$\mu - \mu_0 + \frac{1}{\varepsilon}(\mu^2 - \mu_0^2) = \phi_f, \tag{16}$$

$$\mu = \frac{\varepsilon}{2}\left(-1 \pm \sqrt{1 + \frac{4}{\varepsilon}\left(\mu_0 + \frac{1}{\varepsilon}\mu_0^2 + \phi_f\right)}\right) \sim \begin{cases} \mu_0 + \phi_f & \text{if } \varepsilon \gg \phi_f \\ -\frac{\varepsilon}{2} + \sqrt{\varepsilon\phi_f} & \text{if } \varepsilon \ll \phi_f \end{cases}, \tag{17}$$

where $\varepsilon = \pi\hbar^2 v_F^2 / \left(4\pi e^2 d + \frac{1}{D}\right)$.

If D is large, then considering that $\frac{1}{4}\frac{\hbar v_F}{e^2}\frac{\hbar v_F}{d} \approx 400$ meV $\gg \phi_f$, one has $\varepsilon \gg \phi_f$. This scenario seems to be consistent with the large alternate doping in the experiment.

Supplementary Note 6: Electric field from ferroelectric domains

In this section, we discuss the electric field amplitude outside of the ferroelectric layers. Two cases will be discussed in detail: the case is that the moiré period is small and there is no lattice relaxation and the case is in which there is lattice relaxation, and the triangular AB and BA domains are separated by dislocation networks.

Case 1: moiré period is small, and there is no lattice relaxation

The potential in this case has been well discussed by Zhao et al. [14]. According to the Coulomb theorem, the potential at (R, z) is generated by the charge ρ at (R', z'),

$$V(R,z) = \int \frac{\rho(r(R'),z')}{4\pi\varepsilon_0\sqrt{(R-R')^2+(z-z')^2}} dR'd \tag{18}$$

, where r is the relative displacement of the atoms in the two TMD layers.

Now we expand $f = \frac{1}{4\pi\varepsilon_0\sqrt{(R-R')^2+(z-z')^2}}$ near $z' = 0$,

$$V(R,z) = \int \frac{\rho(r(R'),z')}{4\pi\varepsilon_0} \sum_{n=0}^{\infty} \frac{1}{n!} \frac{\partial^n f}{z'^n}\bigg|_{z'=0} z'^n \, dR'dz' \tag{19}$$

We define $P^{(n)}(r(R')) = \int_{\Delta z/2}^{\Delta z/2} \rho(r(R'),z')z'^n dz'$. Thus,

$$V(R,z) = \int \sum_{n=0}^{\infty} \frac{(-1)^n}{n!} \frac{\partial^n f}{z^n}\bigg|_{z=0} \frac{P^{(n)}(r(R')}{4\pi\varepsilon_0} dR' \tag{20}$$

$P^{(n)}(r(R')$ can be denoted using the parameters in the reciprocal space:

$$V(R,z) = e^{-G|z|} \sum_{n=1}^{\infty} \frac{P^{(n)}(r(R))}{2\varepsilon_0 n!} G^{n-1} sgn(z)^n \tag{21}$$

where $G = \frac{4\pi}{\sqrt{3}b}$ (b is the period of the ferroelectric moiré period).

Since the polarization can be approximated as a sinusoidal function, only the leading term will be kept:

$$V(R,z) = \mathrm{sgn}(z) \frac{P^{(1)}(r(R))}{2\varepsilon_0} e^{-G|z|} \tag{22}$$

The electric field is

$$E(R,z) = \nabla \mathrm{V} = \mathrm{sgn}(-z) \frac{P^{(1)}(r(R))}{2\varepsilon_0 G} e^{-G|z|} \tag{23}$$

At the TMD surface, $z \ll b$. Thus,

$$E(R, z \ll b) = \text{sgn}(-z)\frac{2\pi P^{(1)}(r(R))}{\sqrt{3}b\varepsilon_0} \quad (24)$$

Case 2: The triangular AB and BA domains are separated by dislocation networks.

This is the case that we have studied in this work. In contrast to the special potential profile with a sinusoidal form, the profile can be approximated by a square function. The step function can still be represented as a sum of multiple sinusoidal waves.

$$P(r(R)) = \frac{4}{\pi}\sum_{n=1}^{\infty}\frac{P sin((2n-1)GR)}{2n-1} \quad (25)$$

At the TMD surface, $z \ll b$. Thus

$$E(R, z \ll b) = \frac{4}{\pi}\sum_{n}^{\infty}\text{sgn}(-z)\text{sgn}(-1)^{2n-1}\frac{sin((2n-1)GR)}{2n-1}\frac{2\pi P^{(1)}(r(R))}{2\varepsilon_0 G}$$

$$= \frac{4}{\pi}\sum_{n}^{\infty}\text{sgn}(-z)\text{sgn}(-1)^{2n-1}\frac{sin((2n-1)GR)}{2n-1}\frac{2\pi P}{\sqrt{3}b\varepsilon_0} \quad (26)$$

n	$E_n(R, z \ll b)$ maximum	$E_n(R, z \ll b)(P = 2\ pC/m$, b=100 nm)
1	$\frac{4}{\pi}\frac{2\pi P}{\sqrt{3}b\varepsilon_0}$	10.2 mV/nm
2	0	0
3	$-\frac{4}{5\pi}\frac{2\pi P}{\sqrt{3}b\varepsilon_0}$	-2.0 mV/nm
4	0	0
5	$\frac{4}{9\pi}\frac{2\pi P}{\sqrt{3}b\varepsilon_0}$	1.1 mV/nm
6	0	0
7	$-\frac{4}{13\pi}\frac{2\pi P}{\sqrt{3}b\varepsilon_0}$	-0.8 mV/nm

Supplementary Note 7: Regime with plasmon propagation

Here we analyze the regimes in which graphene is heavily doped by electrons and holes (shaded areas in Fig. 2b). Upon increasing the carrier density, the momentum of dispersive plasmon mode at a particular energy gradually decreases (from $\sim 10/a$ to $\sim 0.1/a$, where a is the tip radius of curvature), following the graphene plasmon scaling rule, $\omega_p/\sqrt{q} \propto \sqrt[4]{n}$. Once the plasmon momentum matches the tip momentum($\sim 1/a$), the plasmon mode can better couple to the tip and produces stronger scattering amplitude[15]. At the same time, this momentum match allows the tip to launch or reflect propagating plasmon polaritons. Likewise, the sharp domain boundaries with a width of ~10 nm can also reflect and launch plasmon polaritons[16]. The constructive and destructive interference of the plasmon results in complicated plasmon patterns, and therefore no regular periodic fringes can be observed. Now the measured scattering amplitude is contributed to by the electrical field of the plasmon polariton, besides the local optical conductivity. Consequently, at high doping, the triangular domains become blurry in the scattering amplitude images, as shown in Fig. 2c, and the back gate shift between the two profiles reduces (Fig. 2b).

Supplementary Note 8: Photon energy dependence of the ferroelectric modulated plasmonic response

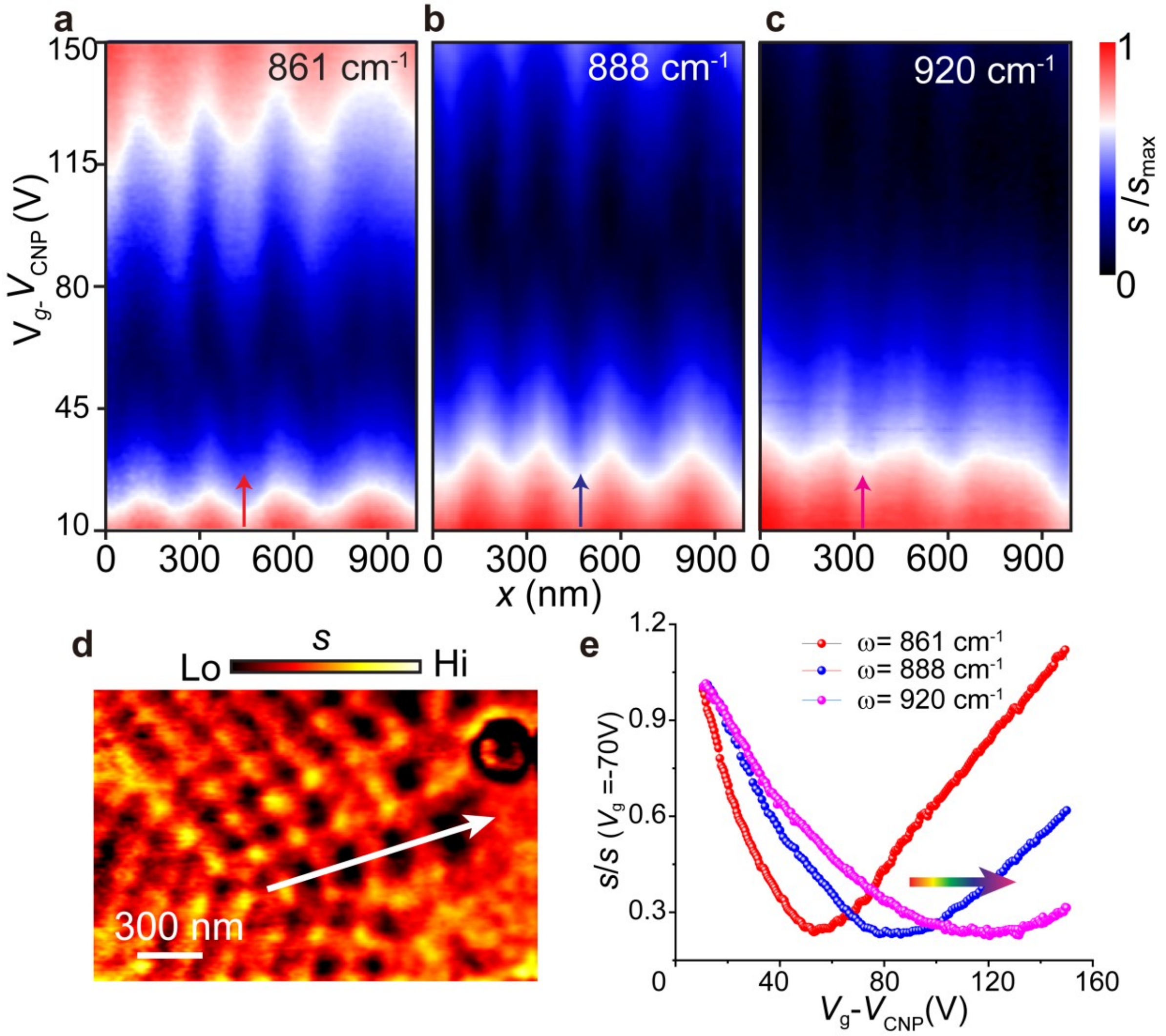

Supplementary Figure 14| Nanoscale optical response of ferroelectric doped graphene as a function of electrostatic doping and excitation energy. a,b,c, The near-field scattering amplitude as a function of gate-voltage $V_g - V_{\mathrm{CNP}}$, measured along a line trace that crosses several domains (marked with the white line in **d**). **d**, Image of the near-field scattering amplitude s_4 at excitation energy ω=888 cm^{-1} and $V_g - V_{\mathrm{CNP}} = 125\ V$. **e**, The evolution of the plasmonic response as a function of global back gate voltage probed at various photon energies. The arrows in panels **a**, **b**, and **c** indicate where the line profiles are taken. As indicated by the arrow in **e**, the plasmonic resonance shifts to higher carrier density with increasing photon energy. All data were acquired on Device A.

In this section, we investigated the photon energy dependence of the ferroelectrically modulated plasmonic response with representative results for Device-A plotted in Supplementary Fig.14a-c. Under various photon energies, infrared spectral features are similar to those described above.

First, the neighboring domains display scattering amplitude contrast, and this contrast reverses when graphene doping is tuned via the back gate. Second, when graphene is being doped away from CNP, the scattering amplitude first decreases to a minimum and then increases. This increased scattering amplitude arises from the better coupling between the plasmon modes and the tip. When the photon energies increase, this coupling occurs at higher carrier doping. To clearly illustrate this dependence, we plot the extracted scattering amplitude line profiles in Supplementary Figure 14e. This finding further confirms that the observed scattering amplitude contrast originates from plasmon excitations as the graphene plasmon energy ω_p, and the carrier density n obey the scaling rule, $\omega_p \propto \sqrt[4]{n}$ (Ref. [4,7,17,18]). In all these data, the near-field amplitude across the domains shows a sinusoid-like profile rather than a step function profile. This spatial feature can be attributed to the carrier density gradient and the gradual transition of near-field amplitude across the domain walls (Supplementary note 9).

To probe the ferroelectric using plasmon response, in principle, the lasers from THz to the middle infrared range can be used. We should select the optimal excitation frequency, based on the plasmonic dispersion and the carrier density of the devices.

Supplementary Note 9: Interpretation of the sinusoidal shape of the near-field profile across domains

In this section, we simulate the near-field amplitude line profiles across domains. Here we assume the domains have a one-dimensional periodic structure and the carrier density distribution across the domains is a rectangular function.

For computational efficiency, we adopt a simplified 2D model using the previously proposed simulation method[19]. In this model, the tip is modeled as a prolate metallic spheroid with a length of 600 nm and an apex radius of curvature of 30 nm. The tip oscillates harmonically above the sample surface with oscillation amplitude $A = 50$ nm. That is, the distance between the tip apex and sample surface is given by $z = A(1 - \cos(\Omega t)) + h_0$, where Ω is the oscillation frequency and $h_0 = 1$ nm is the minimal tip-sample distance.

A schematic of the device and the tip is shown in Supplementary Fig. 15. The sample stack consists of a multiplayer structure, including a 1.4 nm TMD layer, a graphene, a 60 nm hBN, a 285 nm SiO_2, and a Si substrate. At 861 cm^{-1}, the TMD permittivity tensor is given by

$\begin{pmatrix} 12 & 0 & 0 \\ 0 & 12 & 0 \\ 0 & 0 & 4 \end{pmatrix}$. For anisotropic hBN, the permittivity tensor is $\begin{pmatrix} 7.2 + 0.25i & 0 & 0 \\ 0 & 7.2 + 0.25i & 0 \\ 0 & 0 & 2 + i \end{pmatrix}$. SiO_2 has a permittivity of 4. Si has a permittivity of 11.7. The graphene layer consists of alternating domains with periodicity Λ. The carrier density in the lower doping domain is $1 \times 10^{12}\ \text{cm}^{-2}$ while that in the higher doping domain is $1.75 \times 10^{12}\ \text{cm}^{-2}$. The conductivities of the graphene domains are calculated using the well-established Kubo formula[3].

Now the tip position is given by (x, z), where x is the horizontal position. To compute the near-field signal profile directly comparable to the experimental data, we numerically simulate the dipole moment of the tip $p(x, z(t))$ by $p\big(x, z(t)\big) = \int \sigma z dS = \int (\boldsymbol{E} \cdot \boldsymbol{n}) z dS$, where σ is the surface charge density on the tip surface, $\boldsymbol{E}$ is the electric field, and $\boldsymbol{n}$ is the surface normal vector. Finally, the demodulated near-field signal is calculated as $S_n(x) = \int_0^{\frac{2\pi}{\Omega}} p\big(x, z(t)\big) e^{-n\Omega t} dt$.

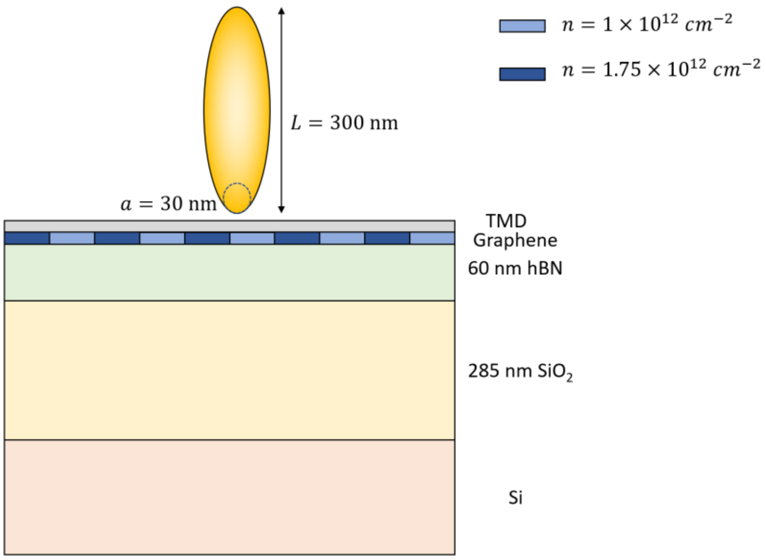

Supplementary Figure 15| Schematic of the simulation setup.

Here we investigate three situations, where $\Lambda = 100$ nm, 200 nm, and 300 nm. The simulated near-field signal profiles are shown in Supplementary Fig. 16. We can see that for domains with small sizes, the near-field profile is reminiscent of a sinusoidal function. When the domain size increases, the near-field profile gradually evolves into a step function. In addition, by comparing s_3 and s_4, we find that the near-field demodulated at a higher harmonic is closer to the step shape[20].

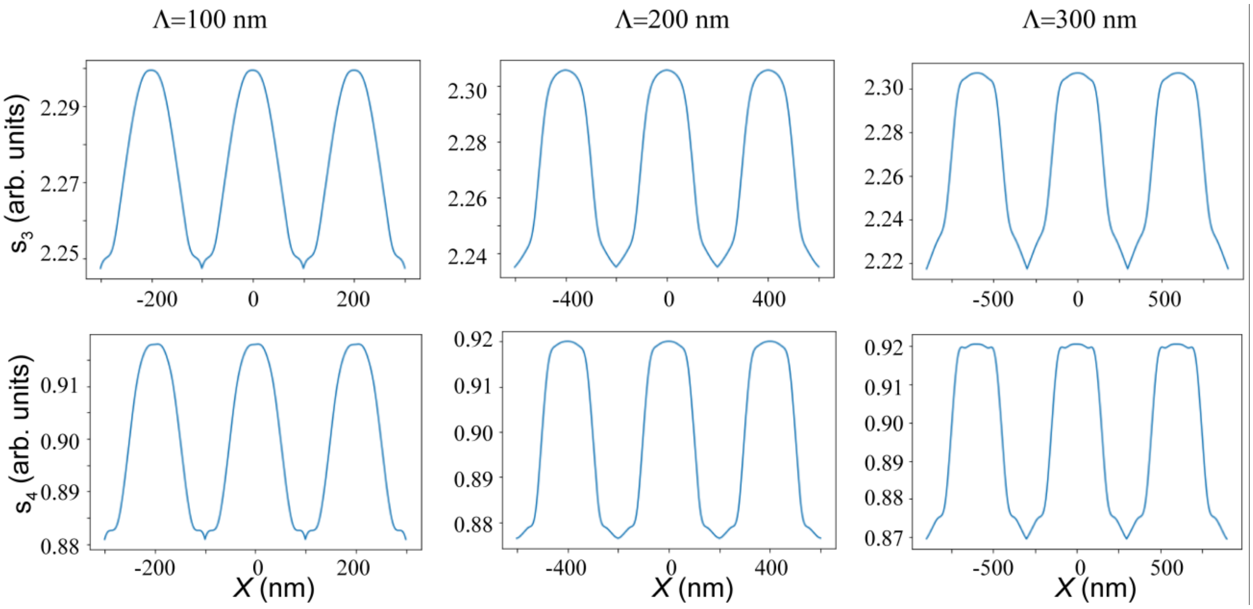

Supplementary Figure 16| Simulated near-field signal profiles as a function of x for three domain sizes.

Supplementary note 10: Quality of the layer interfaces and their effects on ferroelectricity-induced doping

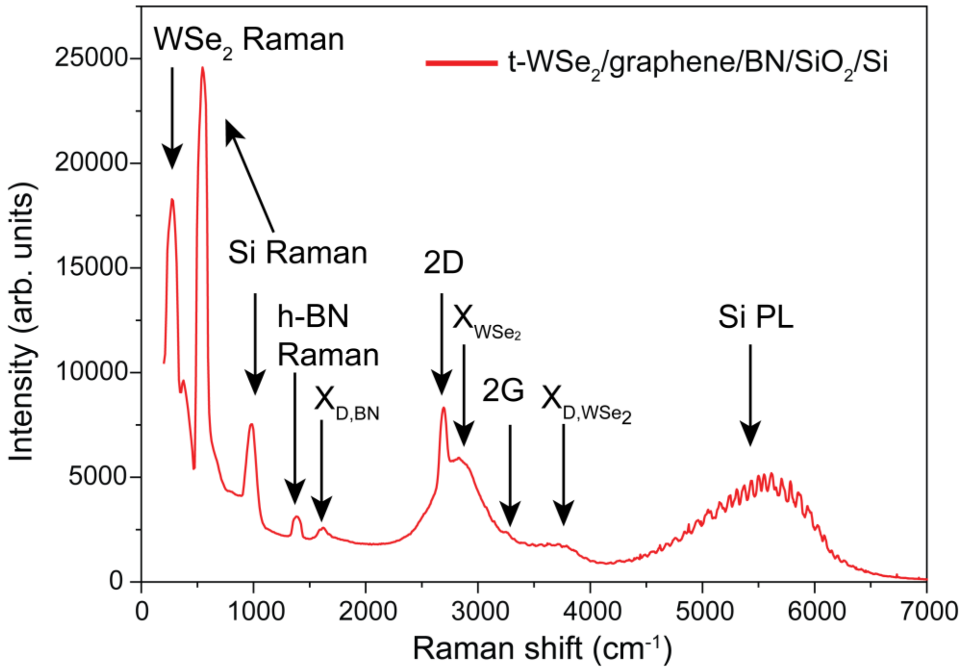

Supplementary Figure 17| Raman spectrum of the ferroelectricity device. A 633 nm excitation wavelength was used. The peaks are assigned as follows: WSe_2 Raman mode: ~270 cm^{-1}; Si Raman modes: 520 and 980 cm^{-1}; h-BN Raman mode: 1380 cm^{-1}; Photoluminescence from h-BN defects ($X_{D,BN}$): ~700 nm (this broad photoluminescence peak buries the graphene G Raman peak); graphene 2D Raman peak (2D): 2700 cm^{-1}; WSe_2 photoluminescence (X_{WSe2}):760 nm; graphene 2G Raman peak (2G): 3250 cm^{-1}; WSe2 photoluminescence from defect ($X_{D,WSe2}$): 820 nm; photoluminescence from Si (Si PL): 980 nm.

To confirm the quality of the stacked devices and check whether there is contamination between the layers, we performed Raman spectrum measurements. As shown in Supplementary Fig. 17, there is not any noticeable Raman signal from organic materials, such as acetone, methanol, polycarbonate (PC), polypropylene (PPC), and polydimethylsiloxane (PDMS), which are used for the sample fabrications. Water is also a possible residual between the stacked layers. However, we did not observe a clear signature of the water Raman peaks.

However, in the spectrum measurements, we observed photoluminescence from defect states of h-BN and WSe_2. The emission peak around 700 nm is from the defect states in h-BN (Ref. [21]). The emission peak around 835 nm originates from the defect excitons in WSe_2 (Ref. [22]).

Supplementary note 11: Electron cooling length in photocurrent measurement

11.1 Electron cooling length

The photocurrent in graphene is dominated by the photo thermoelectric effect. A local current can be formed once a temperature variation occurs at the region with a nonzero Seebeck coefficient gradient, that is $j = \sigma\delta T\nabla S$. Assuming there is a local junction with an inhomogeneous Seebeck coefficient and a local heating source, the photocurrent can be formed if the heating source can increase the electron temperature on the local junction. To generate a nonzero photocurrent, the maximum distance between the local junction and the local heating source (tip in our experiment) is governed by a characteristic length referred to as the electron cooling length. Namely, the cooling length is the scale over which the hot electron generated by the photoexcitation will equilibrate with the substrate.

Assuming that the domains do not influence the thermal diffusion, then in this in-plane isotropic scenario, the temperature profile is $\delta T \propto K_0(x_{tip}/l_{cool})$, where K_0 is the zeroth order modified Bessel function of the second kind, x_{tip} is the distance between the tip and the boundary, and l_{cool} is the cooling length[23]. We examine the photocurrent near a long boundary. Phtocurrent originating from the Seebeck gradient of the long boundary is simply proportional to the increase in electron temperature δT (Ref. [24]). By fitting the photocurrent profile, we find that the cooling length is ~ 600 nm.

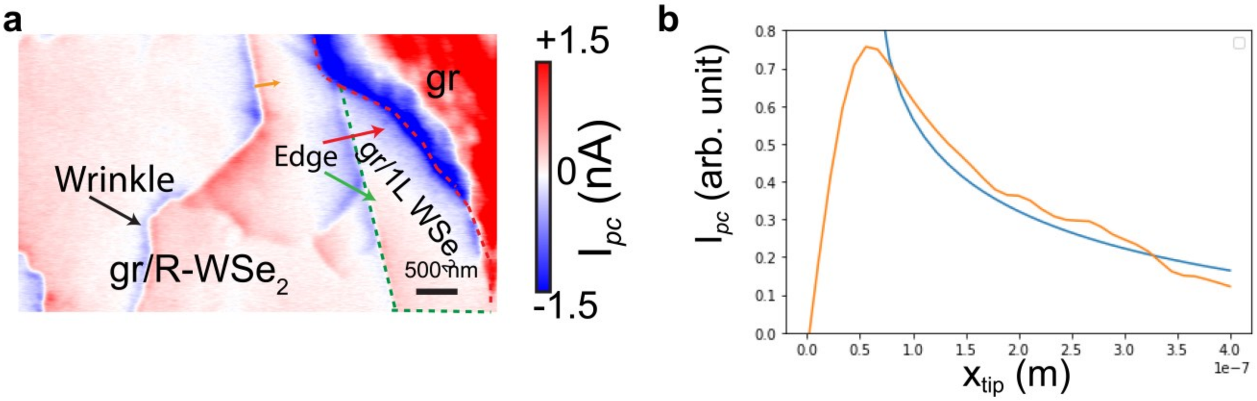

Supplementary Figure 18| Cooling length of the hot electrons. a, Photocurrent mapping of the main text. The winkle in the graphene/R-stacking WSe_2 bilayer (gr/R-WSe_2), the boundary between the gr/WSe2 and gr/1L WSe_2 and the boundary between the gr/1L WSe_2 and graphene are denoted by the black, green and red arrows, respectively. **b,** The line profile of the photocurrent

near the wrinkle. The position is denoted by an orange line with an arrow in **a**. The data are fitted by the blue line.

11.2 Photovoltaic effect in graphene photocurrent

Due to the ferroelectric polarization, a potential difference naturally develops across the domain wall. However, the photocurrent from this potential can be ignored. In our nano-photocurrent experiment, the decay length of photocurrent at the domain wall is hundreds of nanometers, which is much larger than the potential junction width of ~10 nm. The photocurrent originating from the potential at the domain wall is expected to show a fast spatial decay, with a scale of junction width. Conversely, a much slower decay was observed in our nanometer-resolved photocurrent mapping. Our nano-photocurrent results, in concert with previous far-field measurements, corroborate that the photocurrent in graphene is dominated by photothermal effect. Therefore, it is absolutely necessary to introduce the Seebeck effect.

11.3 Spatial scales in the photocurrent measurements

The laser with a wavelength of ~11 um is focused on the sample and tip using a parabolic mirror; the laser spot diameter on the sample is ~30 um. It should be noted that this incident light is locally enhanced by the sharp metallized tip. The nano-photocurrent, acquired by demodulation at the tip-tapping frequency, is induced by the locally enhanced field at the apex of the tip. Therefore, to analyze the nano-photocurrent, the more relevant spatial scale is the size of the locally enhanced field, rather than the laser spot size. This local field is confined to ~ tens of nanometers underneath the tip and is much smaller than the moiré period in this work, which is around hundreds of nanometers.

Supplementary note 12: Near-field photocurrent simulations

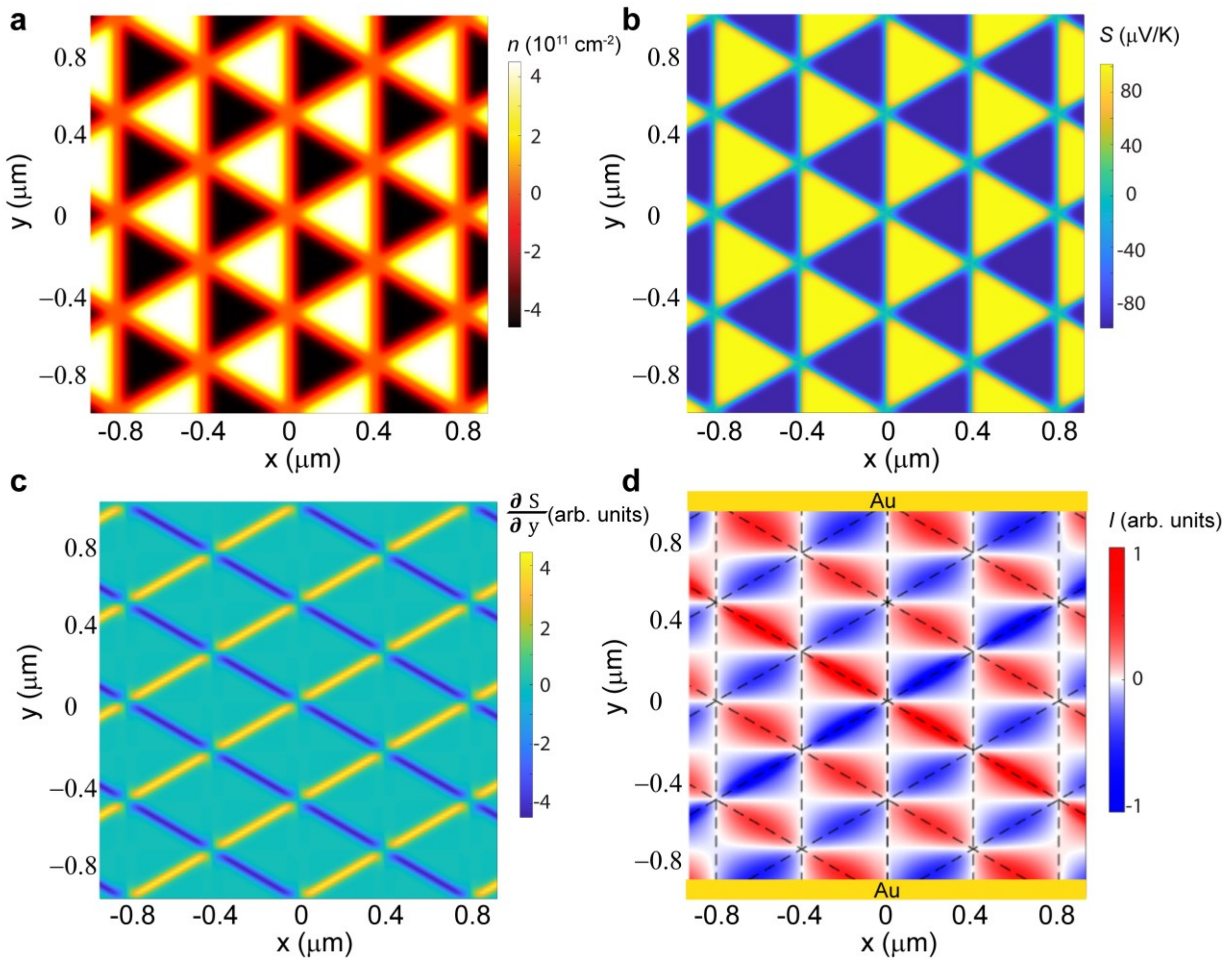

Supplementary Figure 19| Photocurrent simulations with average Fermi energy at CNP. a, b, The checkerboard patterns of the carrier density and the corresponding Seebeck coefficient, respectively. **c,** The gradient of Seebeck coefficient in the y direction. **d,** The photocurrent patterns. The two electrodes are denoted on the top and bottom. In the simulations, we assume that the electrodes are away from the region of interest.

The photocurrent simulation results are consistent with the experimental results in the main text. In the simulation, the auxiliary field is along the y direction. As a result, the domain walls parallel to the y direction do not contribute to the photocurrent. Domains walls along the other two directions possess the opposite gradient of the Seebeck coefficient (Supplementary Fig. 19c), thus forming photocurrent with signal flipping (Supplementary Fig. 19d). The photocurrent peaks at the domain walls, as the Seebeck coefficient gradient reaches its maximum.

To simulate the photocurrent generated by the ferroelectricity-induced charge carrier doping, we implemented a finite element simulation using the Shockley-Ramo theorem[25]. At a high level, the simulation works by first generating the relevant material parameters by using the local carrier density across the sample. We then solve for the auxiliary field of the Shockley-Ramo formalism and calculate the heating of the sample due to the tip at each point in the simulation. Finally, taking all these elements together, we integrate over the whole sample for each point, yielding the photocurrent at that position.

The simulation begins with a rectangular array representing the sample, which we populate with the appropriate charge carrier density ($\pm 3.75 \times 10^{11} \mathrm{cm}^{-2}$) measured from our experiment in a moiré pattern. We assume the carrier density goes as $\mathrm{e}^{-(\frac{x}{L})^2}$ at the domain walls, where x is the distance from the domain wall and $2L$ is the width of the domain wall. We then add an overall constant to this 2D array corresponding to the charge carrier doping due to the back gate voltage, the value of which is determined by the properties of the sample. From this 2D array of carrier densities, we then calculate similar arrays containing the values for the conductivity of the doped graphene and the gradient of the Seebeck coefficient. For the conductivity, we use the results of Ref. [26] as well as the previously calculated carrier density; for the Seebeck Coefficient gradient, we use the Mott Formula.

Next, we solve for the auxiliary field used in the Shockley-Ramo Formalism. The auxiliary field solves the Laplace problem $\nabla \cdot (\sigma(\vec{r})\nabla\psi) = 0$, where $\sigma(\vec{r})$ is the position dependent conductivity. We place a conducting contact with a positive voltage at the top of the sample and a ground contact at the bottom, representing these contacts as boundary conditions with values 1 and 0, respectively. Finally, using MATLAB's PDE solver, we find ψ and more importantly, $\nabla\psi$ on the sample, which is dominated by the gradient in the vertical direction.

Following the calculation of the auxiliary field, we are ready to begin considering individual pixels of the sample to calculate the photocurrent. For each pixel, we take the position of that point as that of the tip. We take the power delivered to the sample to be proportional to the conductivity-dependent absorbance of the graphene. The temperature distribution across the sample is simulated according to the procedure in Ref. [23], and the resulting solution is the zeroth-order modified Bessel function of the second kind scaled by a characteristic cooling length. Having calculated the temperature distribution across the sample for every pixel, we then calculate $I_{PC} =$

$-\int_{\Omega} \sigma \delta T \nabla \psi \cdot \nabla S d^2 \boldsymbol{r}$ for each pixel. Here, σ is the conductivity, δT is the difference between electronic temperature and the equilibrium temperature, $\nabla \psi$ is the gradient of the auxiliary field, and ∇S is the gradient of the Seebeck Coefficient.

Supplementary Reference

1 Fei, Z. *et al.* Gate-tuning of graphene plasmons revealed by infrared nano-imaging. *Nature* **487**, 82-85, doi:10.1038/nature11253 (2012).

2 Woessner, A. *et al.* Highly confined low-loss plasmons in graphene–boron nitride heterostructures. *Nature Materials* **14**, 421-425, doi:10.1038/nmat4169 (2015).

3 Fei, Z. *et al.* Infrared Nanoscopy of Dirac Plasmons at the Graphene–SiO2 Interface. *Nano Letters* **11**, 4701-4705, doi:10.1021/nl202362d (2011).

4 Ju, L. *et al.* Graphene plasmonics for tunable terahertz metamaterials. *Nature Nanotechnology* **6**, 630-634, doi:10.1038/nnano.2011.146 (2011).

5 Li, Z. Q. *et al.* Dirac charge dynamics in graphene by infrared spectroscopy. *Nature Physics* **4**, 532-535, doi:10.1038/nphys989 (2008).

6 Kuzmenko, A. B., van Heumen, E., Carbone, F. & van der Marel, D. Universal Optical Conductance of Graphite. *Physical Review Letters* **100**, 117401, doi:10.1103/PhysRevLett.100.117401 (2008).

7 Wunsch, B., Stauber, T., Sols, F. & Guinea, F. Dynamical polarization of graphene at finite doping. *New Journal of Physics* **8**, 318-318, doi:10.1088/1367-2630/8/12/318 (2006).

8 Falkovsky, L. A. & Pershoguba, S. S. Optical far-infrared properties of a graphene monolayer and multilayer. *Physical Review B* **76**, doi:10.1103/PhysRevB.76.153410 (2007).

9 McLeod, A. S. *et al.* Model for quantitative tip-enhanced spectroscopy and the extraction of nanoscale-resolved optical constants. *Physical Review B* **90**, 085136, doi:10.1103/PhysRevB.90.085136 (2014).

10 Kim, K. *et al.* Band Alignment in WSe2–Graphene Heterostructures. *ACS Nano* **9**, 4527-4532, doi:10.1021/acsnano.5b01114 (2015).

11 Dai, S. *et al.* Graphene on hexagonal boron nitride as a tunable hyperbolic metamaterial. *Nature Nanotechnology* **10**, 682-686, doi:10.1038/nnano.2015.131 (2015).

12 Low, T. *et al.* Plasmons and Screening in Monolayer and Multilayer Black Phosphorus. *Physical Review Letters* **113**, 106802, doi:10.1103/PhysRevLett.113.106802 (2014).

13 Ni, G. X. *et al.* Fundamental limits to graphene plasmonics. *Nature* **557**, 530-533, doi:10.1038/s41586-018-0136-9 (2018).

14 Zhao, P., Xiao, C. & Yao, W. Universal superlattice potential for 2D materials from twisted interface inside h-BN substrate. *npj 2D Materials and Applications* **5**, 38, doi:10.1038/s41699-021-00221-4 (2021).

15 Jiang, B. Y., Zhang, L. M., Castro Neto, A. H., Basov, D. N. & Fogler, M. M. Generalized spectral method for near-field optical microscopy. *Journal of Applied Physics* **119**, 054305, doi:10.1063/1.4941343 (2016).

16 Sunku, S. S. *et al.* Photonic crystals for nano-light in moire graphene superlattices. *Science* **362**, 1153-1156, doi:doi:10.1126/science.aau5144 (2018).

17 Das Sarma, S. & Hwang, E. H. Collective Modes of the Massless Dirac Plasma. *Physical Review Letters* **102**, 206412, doi:10.1103/PhysRevLett.102.206412 (2009).
18 Jablan, M., Buljan, H. & Soljačić, M. Plasmonics in graphene at infrared frequencies. *Physical Review B* **80**, 245435, doi:10.1103/PhysRevB.80.245435 (2009).
19 Chen, X. *et al.* Rapid simulations of hyperspectral near-field images of three-dimensional heterogeneous surfaces – part II. *Opt. Express* **30**, 11228-11242, doi:10.1364/OE.452949 (2022).
20 Mooshammer, F. *et al.* Quantifying Nanoscale Electromagnetic Fields in Near-Field Microscopy by Fourier Demodulation Analysis. *ACS Photonics* **7**, 344-351, doi:10.1021/acsphotonics.9b01533 (2020).
21 Exarhos, A. L., Hopper, D. A., Patel, R. N., Doherty, M. W. & Bassett, L. C. Magnetic-field-dependent quantum emission in hexagonal boron nitride at room temperature. *Nature Communications* **10**, 222, doi:10.1038/s41467-018-08185-8 (2019).
22 Rivera, P. *et al.* Intrinsic donor-bound excitons in ultraclean monolayer semiconductors. *Nature Communications* **12**, 871, doi:10.1038/s41467-021-21158-8 (2021).
23 Sunku, S. S. *et al.* Hyperbolic enhancement of photocurrent patterns in minimally twisted bilayer graphene. *Nature Communications* **12**, 1641, doi:10.1038/s41467-021-21792-2 (2021).
24 Hesp, N. C. H. *et al.* Nano-imaging photoresponse in a moiré unit cell of minimally twisted bilayer graphene. *Nature Communications* **12**, 1640, doi:10.1038/s41467-021-21862-5 (2021).
25 Song, J. C. W. & Levitov, L. S. Shockley-Ramo theorem and long-range photocurrent response in gapless materials. *Physical Review B* **90**, 075415, doi:10.1103/PhysRevB.90.075415 (2014).
26 Song, J. C. W., Rudner, M. S., Marcus, C. M. & Levitov, L. S. Hot Carrier Transport and Photocurrent Response in Graphene. *Nano Letters* **11**, 4688-4692, doi:10.1021/nl202318u (2011).
